# Cantu syndrome–associated SUR2[H60Y] mutation confers selective gain of function on Kir6.1 ATP-sensitive potassium channels

**DOI:** 10.1016/j.jbc.2025.111105

**Published:** 2025-12-23

**Authors:** Jian Gao, Ellen T. Thompson, Colin G. Nichols

**Affiliations:** 1 Department of Cell Biology and Physiology, Washington University in St. Louis, St. Louis, Missouri, USA; 2 Center for the Investigation of Membrane Excitability Diseases, Washington University in St. Louis, St. Louis, Missouri, USA

**Keywords:** Cantu syndrome, K_ATP_ channel, SUR2B, Kir6.1, Kir6.2, DiBAC(4)3, molecular diagnosis

## Abstract

Gain-of-function (GOF) mutations in either Kir6.1 (encoded by *KCNJ8*) or SUR2 (encoded by *ABCC9*) are causally associated with Cantu syndrome (CS), characterized by coarse facial appearance, hypertrichosis, and multiple cardiovascular abnormalities. To date, all SUR2 mutations identified in association with CS have demonstrated GOF because of reduced ATP sensitivity using patch-clamp analysis, with the notable exception of SUR2[H60Y], which showed WT behavior in Kir6.2–SUR2A channels. We readdressed the effect of SUR2[H60Y] on channel function of the relevant Kir6.1–SUR2B channels, in intact cells, in a more physiologically relevant condition using DiBAC4(3) membrane potential measurements. The H60Y mutation uniquely causes a GOF of Kir6.1–SUR2B channels but does not cause GOF in Kir6.2–SUR2B channels. By a chimeric approach, we identify regions of both the very N and C termini of Kir6.1 that are responsible for this effect and further identify a specific residue, valine 334, in Kir6.1, which is necessary for the isoform specificity.

K_ATP_ channels are formed as tetramers of pore-forming subunits, Kir6.1 or Kir6.2, each surrounded by an auxiliary SUR1 or SUR2 subunit (1, 2, 3, 4), which regulates channel function and modulation by nucleotides (5). SUR2 is expressed as two major splice variants, SUR2A and SUR2B (6, 7). K_ATP_ channels expressed in vascular (8), lymphatic (9), and gastrointestinal (10) smooth muscles are composed of Kir6.1 and SUR2B, whereas K_ATP_ channels expressed in cardiac and skeletal muscles (7, 11) are primarily composed of Kir6.2 and SUR2A.

Gain-of-function (GOF) mutations in either Kir6.1 (encoded by *KCNJ8*) (12, 13) or SUR2 (encoded by *ABCC9*) (14, 15) have been causally associated with Cantu syndrome (CS), a rare condition characterized by coarse facial appearance, hypertrichosis, and multiple cardiovascular abnormalities, including patent ductus arteriosus, lymphedema, and cardiac hypertrophy (16). At the molecular level, CS-associated mutations cause K_ATP_ channel GOF by reducing the apparent ATP sensitivity of channel activity, as assessed by inside–out patch clamp assays (13, 14, 17, 18). For CS mutations, Kir6.2–SUR2A channels (rather than Kir6.1–SUR2B) have typically been used to experimentally model the mutational effects because Kir6.2–SUR2A generates stable and spontaneous active currents in the absence of ATP, while spontaneous currents of Kir6.1–SUR2B are typically very small.

To date, all SUR2 mutations identified in association with CS have demonstrated GOF because of reduced ATP sensitivity using this approach, with the notable exception of SUR2[H60Y], which showed WT behavior (19). The patient carrying this mutation presented typical CS phenotypes, supporting a causal association, and a second, unrelated, CS patient with the same mutation was subsequently identified (16). We have recently developed a DiBAC(4)3-based membrane potential assay that can assess K_ATP_ channel activity in intact cells under physiologically relevant conditions (20). In the present study, we have used this assay to readdress the effect of SUR2[H60Y] on channel function of the relevant Kir6.1–SUR2B channels. The data reveal that the mutation uniquely causes a GOF of Kir6.1–SUR2B channels but does not cause GOF in Kir6.2–SUR2B channels. By a chimeric approach, we identified regions of both the N and C termini of Kir6.1, which are responsible for this effect and further identified a specific residue, valine 344 (V334), in Kir6.1 that contributes to the isoform specificity.

## Results

### SUR2[H60Y] hyperpolarizes cells when associated with Kir6.1 but not Kir6.2

**Figure 1:**
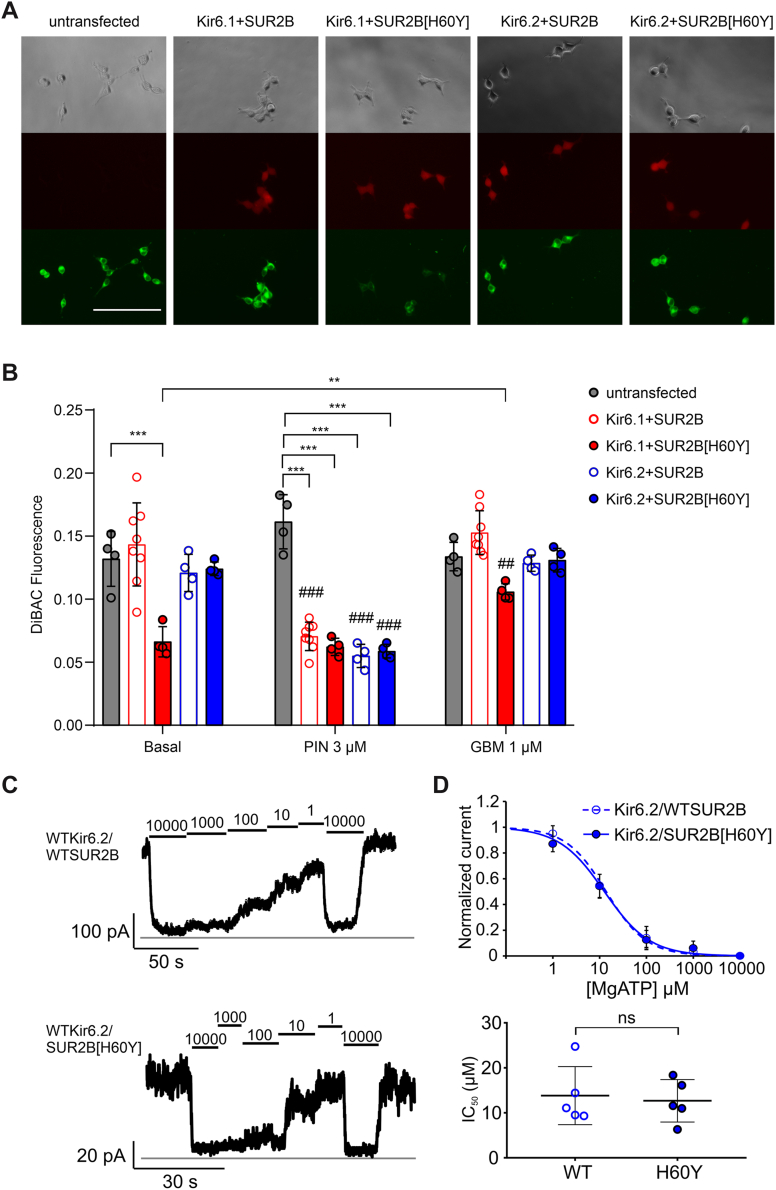
**H60Y induces cell hyperpolarization when associated with Kir6.1 but not with Kir6.2.***A*, representatives of images of stable cell lines incubated with 3 μM DiBAC(4)3 without any other drug treatment. Row 1: phage contrast; row 2: mCherry fluorescence; and row 3: DiBAC fluorescence. The length of the scale bar is 150 μm. *B*, summary of DiBAC(4)3 fluorescence of stable cell lines in the absence or presence of pinacidil (3 μM PIN) or glibenclamide (1 μM GBM). The data points represent individual biological replicates. Two-way ANOVA with Dunnett’s multiple comparisons test. ∗*p* < 0.05, ∗∗∗*p* < 0.001 with comparison as noted. ##*p* < 0.01, ###*p* < 0.001 compared with the corresponding data under basal conditions. *C*, representative traces of currents recorded by inside–out patch-clamp assay in the presence of different concentrations of ATP in the presence of 0.5 mM free Mg^2+^. *D*, summary data of the dose-dependent relationship and the IC_50_ values of recordings in (*C*). Unpaired Student's *t* test. ns *p* ≥ 0.05. ns, nonsignificant.

To assess channel activity in physiological conditions, we measured membrane potential in intact cells stably expressing hKir6.1–hSUR2B or hKir6.1–hSUR2B[H60Y] as well as hKir6.2–hSUR2B or hKir6.2–hSUR2B[H60Y]. DiBAC(4)3 assays were conducted to evaluate the relative membrane potential of the different cell lines (Fig. 1*A*). Under basal conditions, hKir6.1–hSUR2B[H60Y], but none of the other three cell lines, showed decreased DiBAC(4)3 fluorescence compared with untransfected landing pad cells. Specifically, decreased fluorescence of hKir6.1–hSUR2B[H60Y] compared with hKir6.1–hSUR2B indicates a hyperpolarizing effect of the H60Y mutation. Pinacidil hyperpolarizes all four cell lines, and channel inhibitor glibenclamide partially reversed the mutation-induced hyperpolarization (Fig. 1*B*), indicating that the hyperpolarization is dependent on K_ATP_ channel activity. Lack of any effect with hKir6.2-associated cells is consistent with previous patch-clamp assays (19) and implies that the hyperpolarizing, that is, GOF, effect of the mutation H60Y is dependent on association with the Kir6.1 isoform, a unique effect that has not been seen with any other CS-associated SUR2 mutations.

Inside–out patch-clamp assay was conducted to reconfirm key results. Since Kir6.1 currents are difficult to detect in inside–out patches (21), only the effect of SUR2B[H60Y] on Kir6.2 pore-forming channel function was evaluated (Fig. 1*C*). The dose-dependent inhibition effect of MgATP is essentially the same for Kir6.2–WTSUR2B and Kir6.2–SUR2B[H60Y] with no significant difference in IC_50_ values (Fig. 1*D*, Table 1), confirming the results in the DiBAC assay as well as previous reports (19).

**Table 1:** Summary of inside–out patch-clamp results

Ion channel	IC_50_ (μM)	Hill coefficient	Number of patches
Kir6.2/SUR2B	13.8 ± 5.8	1.0 ± 0.2	5
Kir6.2/SUR2B[H60Y]	12.7 ± 4.2	0.9 ± 0.3	5
Kir6.2-A(1)/SUR2B	46.1 ± 18.8	1.0 ± 0.2	7
Kir6.2-A(1)/SUR2B[H60Y]	14.9 ± 6.4	0.7 ± 0.1	4
Kir6.2-A(1)[R325V]/SUR2B	25.0 ± 4.0	1.4 ± 0.2	5
Kir6.2-A(1)[R325V]/SUR2B[H60Y]	63.0 ± 17.1	1.5 ± 0.8	5

### The N terminus and residue V334 of Kir6.1 are mainly responsible for the discrepancy of H60Y GOF toward Kir6.x

**Figure 2:**
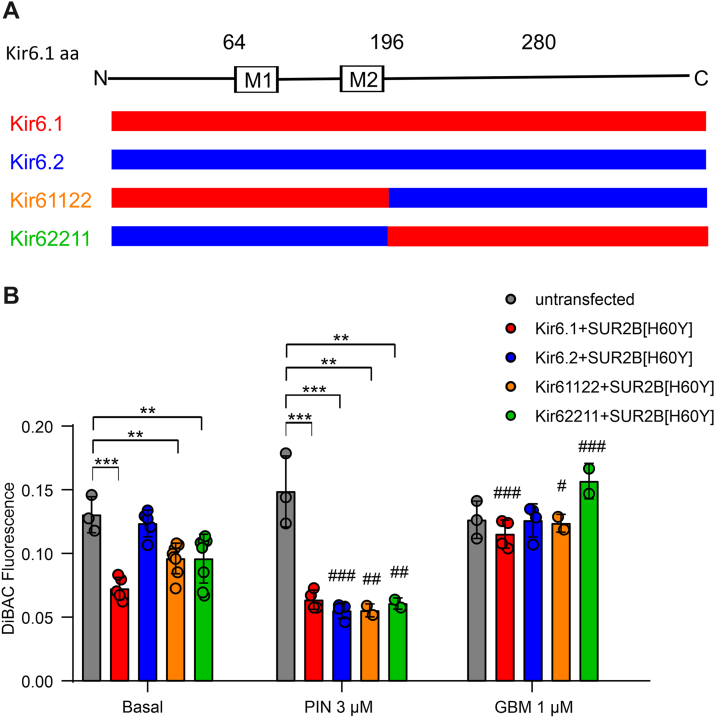
**Two or more sequence elements are responsible for the effect of H60Y on Kir6.1.***A*, scheme of chimera sequences engineered between Kir6.1 and Kir6.2. Chimeras with sequence from Kir6.1 (*red*) and Kir6.2 (*blue*) are shown aligned with the Kir6.1 linear structure. *B*, DiBAC(4)3 fluorescence of untransfected cells and cells expressing Kir6.1, Kir6.2, or their chimeras associated with SUR2B[H60Y] in the absence or the presence of pinacidil (3 μM PIN) or glibenclamide (1 μM GBM). The data points represent individual biological replicates. Two-way ANOVA with Dunnett’s multiple comparisons test. ∗∗*p* < 0.01, ∗∗∗*p* < 0.001 with comparison as noted. #*p* < 0.05, ##*p* < 0.01, and ###*p* < 0.001 compared with the corresponding data under basal condition.

Given the clear difference between Kir6.1 and Kir6.2 in the effect of the SUR2[H60Y] mutation, stable cell lines of a series of chimeric channels were made to isolate key effect-causing regions of the channels. Two chimeric channels were first generated by switching the entire C terminus (∼half the entire subunit sequence) of Kir6.1 and Kir6.2 (referred to as Kir61122 and Kir62211) and coexpressed with SUR2B[H60Y] (Fig. 2*A*). Both Kir61122–SUR2B[H60Y] and Kir62211–SUR2B[H60Y] hyperpolarized cells, but the effects were lower than those of Kir6.1–SUR2B[H60Y]. Both chimeric channels were activated by pinacidil, and their hyperpolarization effect under basal conditions was fully reversed by glibenclamide, indicating otherwise normal function (Fig. 2*B*). These results suggest that both halves of the linear sequence contain residues responsible for the coupling of the H60Y effect to Kir6.1 and that the effects are additive.

**Figure 3:**
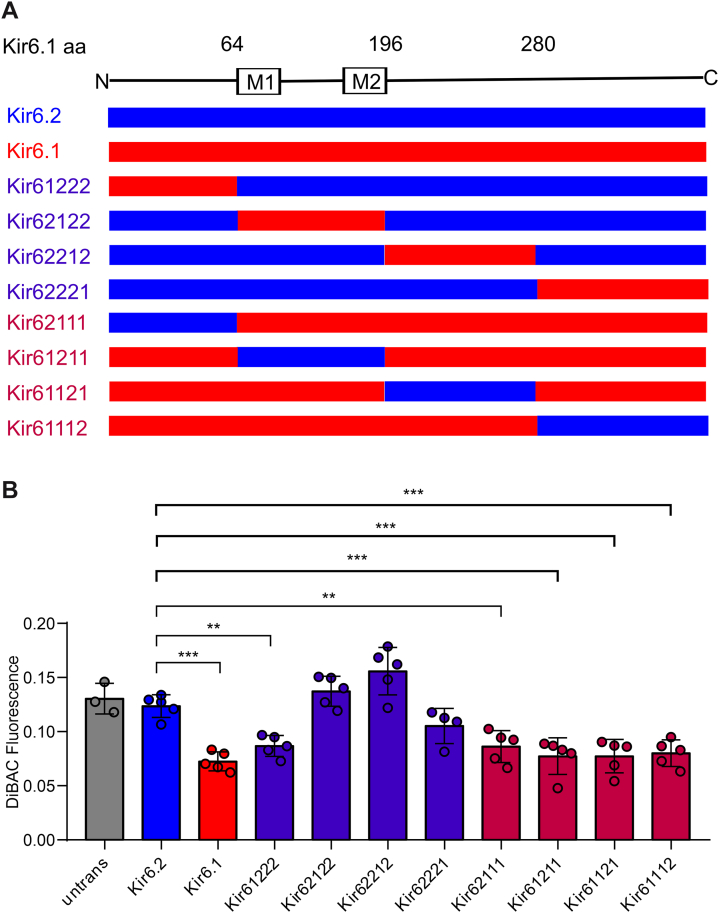
**N and C termini of Kir6.1 are primarily responsible for transducing the activation effect of H60Y.***A*, scheme of chimera sequences engineered between Kir6.1 and Kir6.2. Chimeras with sequence from Kir6.1 (*red*) and Kir6.2 (*blue*) are shown aligned with the Kir6.1 linear structure. *B*, DiBAC(4)3 fluorescence of untransfected cells and cells expressing Kir6.1, Kir6.2, or their chimeras associated with SUR2B[H60Y] under the basal condition. The data points represent individual biological replicates. One-way ANOVA with Dunnett’s multiple comparisons test. ∗∗*p* < 0.01, ∗∗∗*p* < 0.001 with comparison as noted.

Kir6.1 and Kir6.2 sequences were then divided into four segments, namely the whole cytosolic N terminus, the transmembrane domain, and the first and second halves of the cytosolic C terminus (Fig. 3*A*). These constructs were inserted into the Landing Pad cell genome, and *V*_m_ was assessed under basal conditions using the DiBAC(4)3 assay (Fig. 3*B*). Compared with Kir6.2–SUR2B[H60Y], all chimeras except Kir62122–SUR2B[H60Y] and Kir62212–SUR2B[H60Y] showed decreased fluorescence, indicating a relative GOF of the SUR2B[H60Y]-associated channels. A direct interpretation of these results is that both the N terminus and the second half of the C terminus lead to SUR2B[H60Y]-dependent increase of channel activity, albeit the effect of the second half of the C terminus is weaker than that of the N terminus.

**Figure 4:**
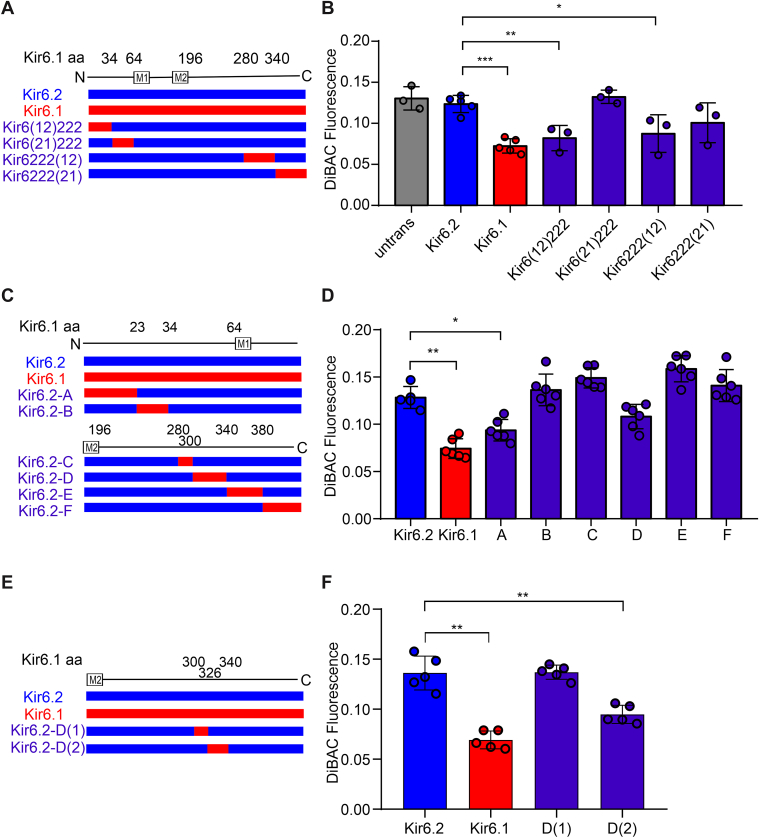
**Further localization of key sequence elements for the H60Y effect.***A*, *C*, and *E*, scheme of chimera sequences engineered between Kir6.1 and Kir6.2. Chimeras with sequence from Kir6.1 (*red*) and Kir6.2 (*blue*) are shown aligned with the Kir6.1 linear structure. *B*, *D*, and *F*, DiBAC(4)3 assay data of stable cell lines expressing SUR2B[H60Y]-associated channels under the basal condition, with chimeras explained in (*A*), (*C*), and (*E*), respectively. The data points represent individual biological replicates. One-way ANOVA with Dunnett’s multiple comparisons test. ∗*p* < 0.05, ∗∗*p* < 0.01, and ∗∗∗*p* < 0.001 with comparison as noted.

**Figure 5:**
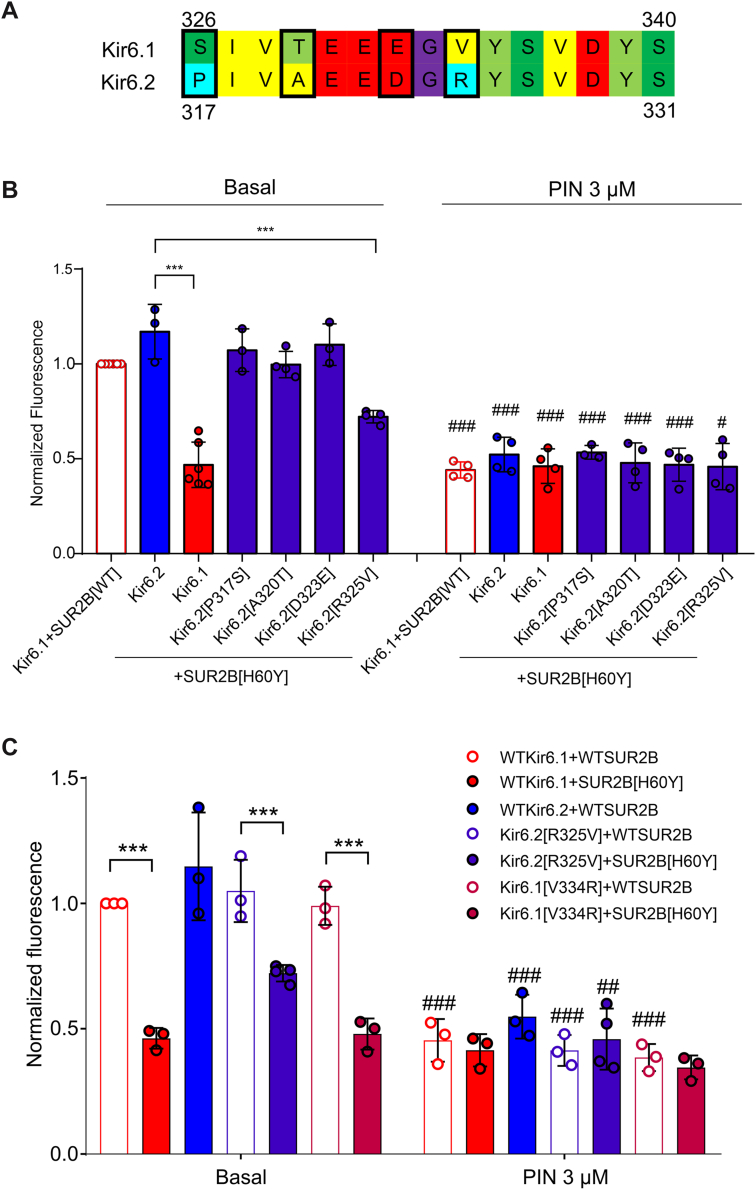
**R325V converts Kir6.2 into an H60Y-sensitive pore subunit.***A*, amino acid sequence alignment of Kir6.1 and Kir6.2 in the corresponding region of D(1). *B*, normalized DiBAC(4)3 data for indicated WT or mutant channel–expressing cell lines under basal conditions or treatment with 3 μM pinacidil. *C*, normalized DiBAC(4)3 data for indicated WT, Kir6.2[R325V], or Kir6.1[V334R] channel–expressing cell lines under basal condition or treatment with 3 μM pinacidil. The data points represent individual biological replicates. One-way ANOVA with Tukey’s multiple comparisons test. ∗∗*p* < 0.01, ∗∗∗*p* < 0.001 with comparison as noted. #*p* < 0.05, ##*p* < 0.01, and ###*p* < 0.001 compared with the corresponding data under basal condition.

The aforementioned process was repeated with additional rounds of generation of increasingly smaller transferred regions from Kir6.1 into Kir6.2 to narrow down the key SURB[H60Y]-coupled sequence elements. For the third-round chimera generation and characterization (Fig. 4, *A* and *B*), three segments, corresponding to chimeras Kir6(12)222, Kir6222(12), and Kir6222(21), were identified as key in providing SUR2B[H60Y]-dependent GOF. A further round of mutagenesis and characterization showed that neither of the two chimeras generated by subdividing the very C-terminal segment from Kir6222(21) (referred to as E and F), hyperpolarized the cells when associated with SUR2B[H60Y], in contrast to the smallest transferred segment from the last but one C-terminal segment (*i.e.*, chimera D, but not C, from further subdivision of Kir6222(12)) (Fig. 4, *C* and *D*). A fifth round of selection further identified chimera D(2), corresponding to amino acids 326 to 340 in Kir6.1 (Fig. 4, *E* and *F*). In this construct, only four amino acids are different between Kir6.1 and Kir6.2, and individual point mutations were then made in the WT hKir6.2 construct, replacing the residue with that of hKir6.1 in the corresponding position (Fig. 5*A*). Of the four individual mutations within the D(1)-related segment (*i.e.*, amino acids 326–340 in Kir6.1 or 317–331 in Kir6.2), only Kir6.2[R325V] successfully generated more active channels (Fig. 5*B*). Cells expressing Kir6.2[R325V]–hSUR2B channels were further generated. Comparison of DiBAC(4)3 fluorescence with Kir6.2[R325V]–hSUR2B[H60Y]-expressing cells confirmed an activation effect of H60Y with the Kir6.2[R325V] pore-forming subunit (Fig. 5*C*). The reverse mutation, Kir6.1[V334R], did not alter the coupling effect of H60Y (Fig. 5*C*). This suggests that V334 is sufficient but not necessary for coupling, and that other residues in Kir6.1 may also convey the coupling effect.

**Figure 6:**
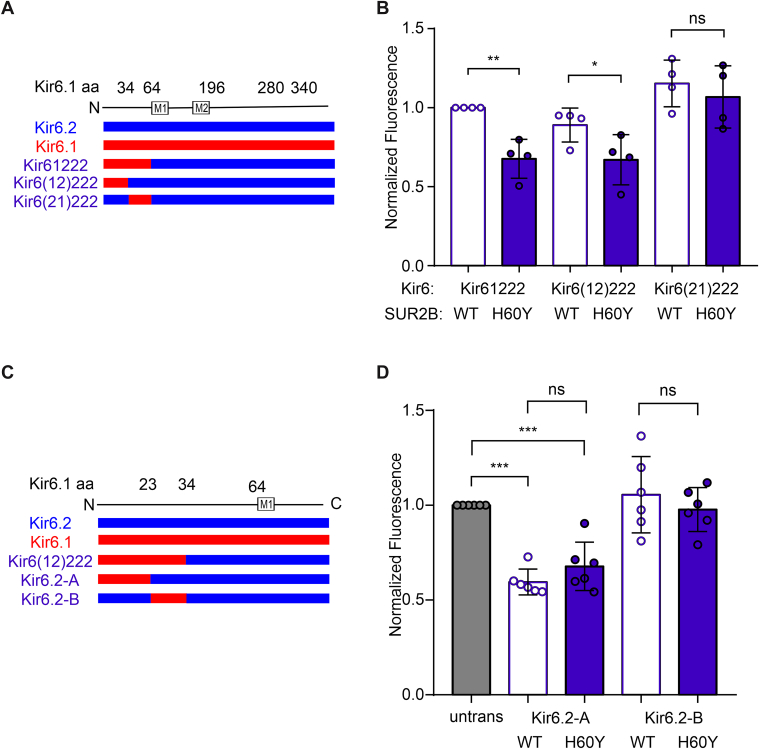
**Further subdivision of the N terminus generates a confounding GOF effect.***A* and *C*, scheme of chimera sequences engineered between Kir6.1 and Kir6.2. Chimeras with sequence from Kir6.1 (*red*) and Kir6.2 (*blue*) are shown aligned with the Kir6.1 linear structure. *B* and *D*, DiBAC(4)3 assay data of stable cell lines under the basal condition, with chimeras explained in (*A*, *C*), respectively. The data points represent individual biological replicates. One-way ANOVA with Tukey’s multiple comparisons test. ∗∗*p* < 0.01, ∗*p* < 0.05, ∗∗∗*p* < 0.001, ns *p* ≥ 0.05 with comparison as noted. GOF, gain of function; ns, not significant.

The N-terminal Kir6(12)222 chimera also generated channels that showed SUR2B[H60Y]-dependent hyperpolarization (Fig. 6, *A* and *B*). Further subdivision of the N-terminal sequence to generate chimeras A and B resulted in strong hyperpolarization of the A construct with both WT SUR2B and SUR2B[H60Y] but no obvious effect of the B construct (Fig. 6, *C* and *D*). No significant effect was caused by the H60Y mutation, obviating further dissection of relevant sequence elements. Since neither segment (residues 1–23 and residues 24–34) in Kir6.1 transduced the coupling effect of SUR2B[H60Y] independently, even though the combined segment (residues 1–34) does, it is conceivable that more than one residue within the combined segment is required to work together to couple the H60Y effect.

### Electrophysiological confirmation of the effect of Kir6.2[R325V]

**Figure 7:**
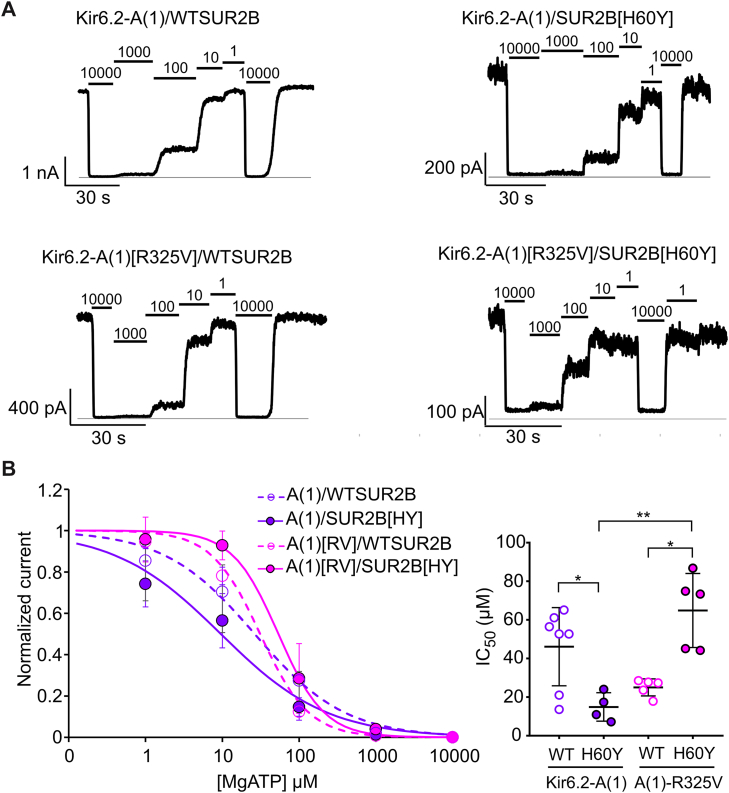
**Mutation R325V couples the gain-of-function effect of SUR2B[H60Y] in Kir6.2-A(1) chimera.***A*, representative inside–out patch-clamp recording traces of Kir6.2-A(1) or Kir6.2-A(1)[R325V] associated with WTSUR2B or SUR2B[H60Y] in the presence of different concentrations of ATP in the presence of 0.5 mM free Mg^2+^. *B*, summary data of the dose-dependent relationship and the IC_50_ values of recordings in (*A*). The data points represent individual biological replicates. One-way ANOVA with Games–Howell’s multiple comparisons test. ∗*p* < 0.05, ∗∗*p* < 0.01.

To directly confirm the ability of Kir6.2[R325V] to confer an activating effect on Kir6.2-dependent channels, we attempted recordings of Kir6.2[R325V]–WTSUR2B and Kir6.2[R325V]–SUR2B[H60Y] channels, but spontaneous currents were too small to reliably assess nucleotide dependence. We switched to study the effect of Kir6.2[R325V] in the Kir6.2-A(1) chimera, in which the N-terminal 17 amino acids are replaced by the Kir6.1 sequence. When coexpressed with SUR2B, this chimera expressed large patch currents, and the dose-dependent effect of MgATP on Kir6.2-A(1)–WTSUR2B, Kir6.2-A(1)–SUR2B[H60Y], Kir6.2-A(1)[R325V]–WTSUR2B, and Kir6.2-A(1)[R325V]–SUR2B[H60Y] was measured(Fig. 7*A*). SUR2B[H60Y] decreased the channel activity of Kir6.2-A(1) by making the channel less resistant to MgATP inhibition, but SUR2B[H60Y] increased the channel activity in the presence of R325V in Kir6.2 (Fig. 7*B*, Table 1). Viewed alternatively, these results show that R325V has no effect on the ATP sensitivity of channels coexpressed with WTSUR2B but has considerably lower ATP sensitivity when coexpressed with SUR2B[H60Y] (Fig. 7*B*, Table 1).

## Discussion

*In vitro* characterization of the effect of a disease-associated mutation in a particular protein is important in two aspects. First, it helps with building a connection between a disease and a particular protein, especially when the molecular or cellular mechanism of the disease is unclear. Second, of clinical relevance, it can provide a more precise diagnosis by providing definitive evidence of a pathogenic effect or a benign effect on protein function itself. For CS, with the single exception of SUR2[H60Y], all studies to date have demonstrated a clear molecular GOF in recombinant K_ATP_ channels generated by identified associated variants in Kir6 or SUR2 subunits, firmly establishing the connection between CS and such molecular defects, and beginning to establish genotype–phenotype correlation (12, 13, 14, 15, 17, 18, 20). It is therefore quite striking that SUR2[H60Y] failed to generate a GOF in studies of Kir6.2–SUR2A channels (19). By establishing that the effect is Kir6 isoform specific, the present study resolves the issue, showing a clear GOF in the CS-relevant Kir6.1–SUR2B channels but not in Kir6.2–SUR2B channels.

### Novel SUR–Kir6 interactions uncovered by SUR2[H60Y]

**Figure 8:**
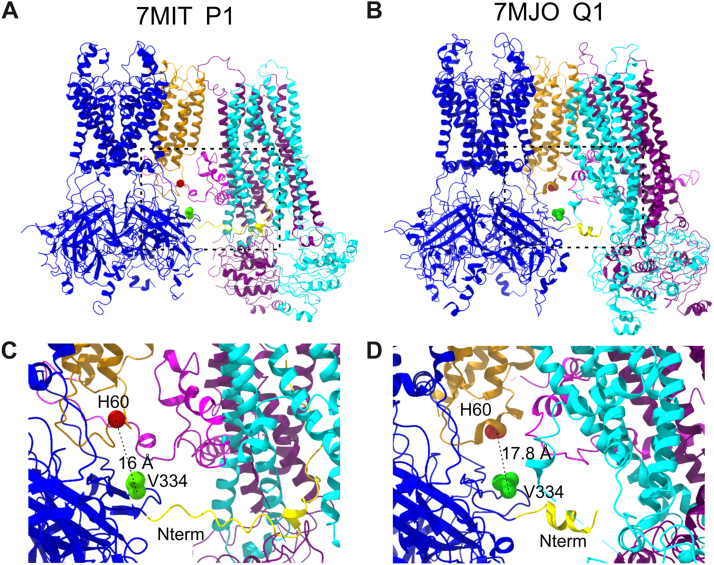
**Localization of His60 in SUR2B and Val334 in Kir6.1.** Structures of Kir6.1–SUR2B in propeller (*A*) and quatrefoil conformations (*B*) (4). Residues of His60 in SUR2 and Val334 in Kir6.1 are shown as *red* and *green spheres*, respectively, with the Cα–Cα distances noted for propeller (*C*) and quatrefoil (*D*) conformations. The N terminus of Kir6.1 is shown as a *yellow ribbon*. Val334, valine 334.

This study is based on the surprising and unprecedented finding that the H60Y mutation in SUR2B causes a GOF in K_ATP_ channels containing Kir6.1, but not Kir6.2, pore-forming subunits. By generating chimeric subunits between Kir6.1 and Kir6.2, we localized two regions in Kir6.1 that are responsible for the Kir6.1-dependent GOF, one located at the N terminus of Kir6.1 and the other in a single residue V334. High-resolution Kir6.1–SUR2B structures (4) suggest that neither the first 17 amino acids nor V334 of Kir6.1 are physically close enough to His60 in SUR2B to interact directly, at least in the “propeller” and “quatrefoil” closed channel conformations with glibenclamide bound in SUR2B (Protein Data Bank codes: 7MIT and 7MJO) (Fig. 8). A previous study identified a unique residue pair, Glu203 from SUR1 and Gln52 from Kir6.2, which can significantly affect channel nucleotide sensitivity (22), at a distance from the nucleotide binding sites, and such an allosteric effect might underlie the unique interaction uncovered here because of the close distance between the residue pair and His60 in SUR2. Isoform-specific mutant effects have been observed in other heteromeric channels. For instance, KCNE1[A8V] reduces current amplitude when associated with KCNH2 but not KCNQ1, whereas KCNE1[R98W] reduces current amplitude when coexpressed with KCNQ1 but not KCNH2 (23). Also, β1[R60C] shifts both activation V_0.5_ and inactivation V_0.5_ of Nav1.2 and Nav1.6 but not Nav1.1 to the depolarized direction (24). However, the mechanism of such auxiliary subunit mutation-caused selective effect on pore-forming subunits is unknown. The Arg60 of β1 is located within the extracellular β-sheet region, which has no direct contact with any observable regions of the pore-forming α subunit, similar to what we discovered in this article.

Finally, while the SUR2[H60Y] effect is thus far unprecedented in terms of a disease-relevant K_ATP_ isoform–specific effect, it may not be unique, given that interaction contacts between Kir6 and SUR subunits are likely to change with isoform pairing structures (4).

### Reanalysis of the phenotypes of the two patients with SUR2B[H60Y]

To date, two unrelated CS patients have been reported carrying the H60Y mutation. One is a female patient originally described as patient 1 in a report in 2011 (25), and the mutation reported subsequently (14); the other is a male patient, reported in 2019 (16). Typical CS features, including congenital hypertrichosis, joint laxity, heart enlargement, patent ductus arteriosus, and edema, were all recorded in both patients (Table 2). The GOF effect of the mutation in the Kir6.1–SUR2B channel correlates well with these clinical records, and hence, the straightforward conclusion is that SUR2B[H60Y] is the cause of the CS phenotypes of the two patients. Since the mutation hyperpolarizes only cells expressing Kir6.1-associated, but not Kir6.2-associated, channels, it can be inferred that GOF in Kir6.1-associated channels and not Kir6.2-associated channels is enough to cause these reported CS symptoms. By extension, it might also be inferred that CS phenotypes that are not observed in these two patients might therefore result from Kir6.2–SUR2 GOF, and since this subunit makeup is most prominent in striated muscle, muscle phenotypes might be absent from SUR2[H60Y] patients. Indeed, hypotonia, found in many CS patients, was absent in the patient reported by Grange *et al.* (16), although delayed motor development was ascribed to low muscle tone and joint laxity in the patient reported by Scurr *et al.* (25). Further pathophysiological relevance could be potentially revealed by future clinical assessment of SUR2[H60Y] patients or by generation of an animal model with SUR2[H60Y] introduced.

**Table 2:** Phenotypes of patients with H60Y mutation

Phenotypes	Polyhydramnios	Congenital hypertrichosis	Wrinkled and/or loose skin	Skeletal dysplasia	Joint laxity	Breathing difficulties
Patient 1	(+)	(+)	(+)	(+)	(+)	(+)
Patient 2	NA	(+)	(+)	NA	(+)	(+)
Phenotypes	Developmental delay	Osteopenia/osteoporosis	Scoliosis	Pectus carinatum	Hypotonia	Exercise intolerance
Patient 1	(+)	(−)	(−)	(−)	(−)	(−)
Patient 2	(+)	NA	NA	(+)	(−)	(+)
Phenotypes	Hernia	Intestinal dysmotility	Patent ductus arteriosus	ASD	VSD	Patent ductus arteriosus surgery/age
Patient 1	(−)	(−)	(+)	(−)	(−)	+/1 year
Patient 2	NA	(+)	(+)	(−)	(−)	+/1 month
Phenotypes	Aortic root dilation/age of diagnosis	Enlarged heart/age of diagnosis	Pericardial effusion/treatment	Arrhythmia	Pulmonary hypertension/age of diagnosis	Low blood pressure
Patient 1	(−)	+/1 month	(−)	(−)	(−)	(−)
Patient 2	(−)	+/0 months	+/0	(+)	(−)	(−)
Phenotypes	High blood pressure	Lymphedema	Abnormal blood vessels	Edema/treatment	Other cardiac defects	Cardiac medications
Patient 1	(−)	(−)	(−)	+/Other diuretics	Patent foramen ovale	(−)
Patient 2	(−)	(−)	NA	+/Spironolactone furosemide	(−)	Metoprolol
Phenotypes	Headaches/migraine	Headache medication	Seizures/type of seizures	Seizure medication	Brain abnormalities	Hemiparesis
Patient 1	(−)	(−)	(−)	(−)	(−)	(−)
Patient 2	Migraine	NA	(+)	NA	NA	NA
Phenotypes	Stroke/age					
Patient 1	(−)					
Patient 2	NA					

NA, the clinical data are not available.

## Experimental procedures

### Molecular biology and cell culture

The identified H60Y variant was introduced into human SUR2B (20) (pcDNA3.1-_ hSUR2B; GenBank TM accession no.: NP_064693.2) complementary DNA using site-directed mutagenesis and verified by direct Sanger sequencing. WT and H60Y hSUR2B complementary DNA were subcloned into a vector including hKir6.x (or related chimeras) and IRES sequence for stable cell line generation using the landing pad human embryonic kidney 293 cell system (26). hKir6.x chimeras (in attB-containing vector) were made by transformation of competent *E. coli* bacteria with two fragments of DNA with homology overlaps at both ends, one in the origin region of the vector covered by the forward primer: CTTCGGAAAAAGAGTTGGTAGCTCTTGATCCG GCAAAC and reverse primer: GTTTGCCGGATCAAGAGCTACCAACTCTTTTTCCGAAG, the other at the junction of the Kir6.1 and Kir6.2 sequences. For the chimera Kir61222, the forward sequence is TTCACCACCTTGGTGGACCTCAAGTGGCCACACACAT, and the reverse sequence is GAGGTCCACCAAGGTGGTGAAGATGTCCTGTAGAA, with ∼20 bp reverse complementary sequence (underlined).

Boundaries of the chimeras used in this article are as follows: Kir61222: Kir6.1[^61^FTTL^64^]–Kir6.2[^64^VDLK^67^]; Kir62111: Kir6.2[^60^FTTL^63^]–Kir6.1[^65^VDLK^68^]; Kir61122: Kir6.1[^192^IFSR^195^]–Kir6.2[^186^HAVI^189^]; Kir62211: Kir6.2[^182^IFSK^185^]–Kir6.1[^196^HAVI^199^]; Kir61112: Kir6.1[^277^LYDI^280^]–Kir6.2[^271^APSD^274^]; Kir62221: Kir6.2[^267^LYDL^270^]–Kir6.1[^281^SATD^284^]; Kir6(12)222: Kir6.1[^31^LPKA^34^]–Kir6.2[^34^RFVS^37^]; Kir6(21)222: Kir6.2[^30^QRRA^33^]–Kir6.1[^35^RFIA^38^]; Kir6222(12): Kir6.1[^337^VDYS^340^]–Kir6.2[^332^KFGN^335^]; Kir6222(21): Kir6.2[^328^VDYS^331^]–Kir6.1[^341^KFGN^344^]; Kir6.2-A: Kir6.1[^20^ENLR^23^]–Kir6.2[^23^KPRY^26^]; Kir6.2B: Kir6.2[^20^EDPA^22^]–Kir6.1[^24^KPRI^27^]; Kir6.2-C: Kir6.1[^297^EGVV^300^]–Kir6.2[^292^ETTG^295^]; Kir6.2-D: Kir6.2[^288^EGVV^291^]–Kir6.1[^301^ETTG^304^]; Kir6.2-E: Kir6.1[^377^QNSL^380^]–Kir6.2[^369^RKRS^372^]; Kir6.2-F: Kir6.2[^365^RGPL^368^]–Kir6.1[^381^RKRN^384^]; Kir6.2-A(1): Kir6.1[^14^LARI^17^]–Kir6.2[^18^AEDP^21^]; Kir6.2-D(1): Kir6.1[^322^HRFV^325^]–Kir6.2[^317^PIVA^320^]; Kir6.2-D(2): Kir6.2[^313^QRFV^316^]–Kir6.1[^326^SIVT^329^].

Human embryonic kidney 293 cells stably expressing hKir6.x (or related chimeras) and hSUR2B or hSUR2B[H60Y] were generated as described previously (20). Cells were cultured with Dulbecco’s modified Eagle’s medium supplemented with 10% fetal bovine serum, 100 U/ml penicillin, 0.1 mg/ml streptomycin, 2 μg/ml doxycycline, and 1 μM puromycin.

### Membrane potential assessments by voltage-sensitive fluorescent dye

These experiments were conducted as described previously (20) with minor adjustments. Briefly, cells in each well were cultured with 100 μl Dulbecco’s modified Eagle’s medium containing 10% fetal bovine serum, 100 U/ml penicillin, 0.1 mg/ml streptomycin, 2 μg/ml doxycycline, and poly-l-lysine (0.002 mg/ml) (27) with appropriate density (∼50–200 cells per vision in the final images). After 1 day in culture, the medium was discarded, and the cells were washed twice with low [K] (139 mM NaCl, 1 mM KCl, 2 mM CaCl_2_, 1 mM MgCl_2_, 10 mM Hepes, 10 mM glucose, pH adjusted to 7.4 with 1 mM NaOH) buffer (80 μl/well). Cells were then incubated with 3 μM DiBAC4(3) in low potassium buffer, with or without additional channel modulators, for at least 20 min. Separate images of green and red fluorescence were taken with the EVOS M5000 imaging system (10× lens). But for a better presentation in Figure 1, images were taken with a 20× lens. Exported TIFF images were analyzed by CellProfiler with custom-designed programs for landing pad cells and generated stable cell lines. Multiple measurements of each cell were exported as CSV format files, and the intensities of all cells as well as the mean cell value in each image were extracted with a custom script written in Python. The average intensity value of duplicate images was calculated as one data point for the statistical analysis and data presentation.

### Excised inside–out patch-clamp experiments

Stable cell lines were trypsinized ∼1 h before the experiment and plated onto the cover slips with medium containing 2 μg/ml poly-l-lysine to increase the cell attachment (27). Pipettes were made from soda lime glass microhematocrit tubes (Kimble) and had a resistance of 1 to 3 MΩ when filled with pipette solution. The bath and pipette solutions (KINT) contained (in millimolar):140 KCl, 10 Hepes, 1 EGTA (pH = 7.4 with KOH). Currents were recorded at a constant holding a potential of −50 mV in the absence and presence of nucleotides, as indicated. Free Mg^2+^ concentrations were maintained at 0.5 mM by supplementation of MgCl_2_ as calculated using https://somapp.ucdmc.ucdavis.edu/pharmacology/bers/maxchelator/webmaxc/ webmaxcS.htm. Rapid solution exchange was attained using a Dynaflow Resolve perfusion chip (Cellectricon). Experiments were performed at 20 to 22 °C. K_ATP_ channel currents in solutions of varying nucleotide concentrations were normalized to the basal current in the absence of nucleotides, and dose–response data were fit with a four-parameter Hill fit according to Equation 1, using the Data Solver Function in Microsoft Excel:

(1) $$\text{Normalized}\;\text{current}=I_{\min}+\left(I_{\max}-I_{\min}\right)/\left(1+{\left(\left[X\right]/IC_{50}\right)}^{H}\right)$$

where the current in KINT = *I*_max_ = 1; *I*_min_ is the normalized minimum current observed in MgATP; [X] refers to the concentration of MgATP; IC_50_ is the concentration of half-maximal inhibition; and H denotes the Hill coefficient.

### Data analysis

All statistical analyses were performed using Microsoft Excel or Prism (GraphPad). Significance values were calculated using one-way or two-way ANOVA, or Student's *t* test, as appropriate, and indicated in figure legends. All values are expressed as mean ± SD.

## Data availability

The original data will be made available to any interested parties upon reasonable request.

## Conflict of interest

The authors declare that they have no conflicts of interest with the contents of this article.
